# Felid Herpesvirus Type 1 Infection in Cats: A Natural Host Model for Alphaherpesvirus Pathogenesis

**DOI:** 10.5402/2012/495830

**Published:** 2012-11-14

**Authors:** Roger Maes

**Affiliations:** Departments of Pathobiology and Diagnostic Investigation and Microbiology and Molecular Genetics, College of Veterinary Medicine, Michigan State University, East Lansing, MI 48824, USA

## Abstract

Feline herpesvirus 1 (FeHV-1) is an alphaherpesvirus that causes feline viral rhinotracheitis, an important viral disease of cats on a worldwide basis. Acute FeHV-1 infection is associated with both upper respiratory and ocular signs. Following the acute phase of the disease lifelong latency is established, primarily in sensory neuronal cells. As is the case with human herpes simplex viruses, latency reactivation can result in recrudescence, which can manifest itself in the form of serious ocular lesions. FeHV-1 infection in cats is a natural host model that is useful for the identification of viral virulence genes that play a role in replication at the mucosal portals of entry or are mediators of the establishment, maintenance, or reactivation of latency. It is also a model system for defining innate and adaptive immunity mechanisms and for immunization strategies that can lead to better protection against this and other alphaherpesvirus infections.

## 1. Introduction

Felid herpesvirus 1 (FeHV-1) is classified under the Order: *Herpesvirales*, Family: *Herpesviridae*, Subfamily: *Alphaherpesvirinae*, and genus: *Varicellovirus* [1]. Characteristics of the members of the *Alphaherpesvirinae* are their short replication cycle, induction of lifelong latency, primary in neurons, and, in most cases, a narrow host range. Both human and animal herpesviruses are members of the *Alphaherpesvirinae *subfamily. Human herpes simplex viruses types 1 (HSV-1) and 2 (HSV-2), respectively, cause cold sores and genital lesions. Varicella zoster virus (VZV) is the causative agent of chickenpox, and the reactivation of latent VZV DNA causes shingles. Some of the mammalian herpesviruses, besides FeHV-1, classified under this family include bovine herpesvirus-1 (BoHV-1), which causes respiratory disease and abortions in cattle, equine herpesvirus-1 (EHV-1), which causes respiratory disease, abortions, and in some cases neurological disease in horses, Suid herpesvirus 1, also known as pseudorabies (PRV) and Aujeszky's disease virus, leading to respiratory disease, abortions, neurological disease in swine, and canid herpesvirus-1 (CaHV-1), responsible for neonatal mortality in puppies and also respiratory and ocular disease in juvenile and mature dogs. Examples of avian alphaherpesviruses are infectious laryngotracheitis virus (ILTV), causing severe respiratory disease in poultry, and Marek's disease virus (MDV), which induces immunosuppression and T-cell lymphomas.

FeHV-1 infection causes feline viral rhinotracheitis (FVR), which not only accounts for approximately half of all diagnosed feline viral upper respiratory infections, but is also an important cause of ocular lesions in cats. As is the case for other alphaherpesvirus infections, the acute phase of FVR is followed by lifelong latency. During the latent stage, viral FeHV-1 DNA persists in episomal form, primarily in the nuclei of sensory ganglion neurons. The transcription of viral RNA is very limited, and infectious virus is not produced. The reversal of the latent state, induced by natural stressors or the administration of corticosteroids, can induce viral reactivation in latently infected cells, leading to renewed production of infectious virus. Reactivated infectious virus then travels to the periphery by anterograde axonal transport, potentially leads to clinical signs (recrudescence), and can lead to viral transmission [2–5].

Since FeHV-1 is a primary pathogen of cats, with respiratory and ocular disease components that are similar to those of human herpesviruses, and latency which is easily reactivated under natural conditions, FeHV-1 infection in cats is considered to be a good natural host model to study the comparative molecular pathogenesis of acute and latent alphaherpesvirus infections and to test novel immunization strategies. 

## 2. Virus Characteristics

The size of FeHV-1 virions ranges from 120 to 180 nm. They are composed of a core containing the double-stranded viral DNA genome, an icosahedral capsid surrounding the core, a tegument layer surrounding the capsid, and a lipid bilayer envelope from which glycoprotein spikes are protruding [6, 7].

FeHV-1 primarily infects domestic cats, but lions and cheetahs are also susceptible [3, 8]. *In vitro*, FeHV-1 replicates only in cells of feline origin. Alphaherpesviruses that are genetically related to FeHV-1 are canid herpesvirus 1 (CaHV-1) and phocid herpesviruses(PhHV) 1 and 2 [3, 9–11]. 

## 3. Genomic Organization

Our laboratory [12] reported the first *Sal *I map of the genome of C-27 strain of FeHV-1 and determined that its size was approximately 134 kb. Grail et al. [13] subsequently mapped the genome of the FeHV-1 B927 strain and determined that its genome was only 126 kb in size. The genomic organization of both of these FeHV-1 strains was found to be similar to that of other varicelloviruses. Basically, the FeHV-1 genome consists of two segments of unique DNA, referred to as the Unique Long (UL) and Unique Short (US) regions. The US region of the genome is flanked by a pair of identical, but inverted sequences designated the Internal Repeat Short (IRS) and Terminal Repeat Short (TRS).

We recently reported the first complete genomic sequence of FeHV-1, as well as the construction and characterization of a BAC clone containing the entire viral genome. Complete genomic sequences were derived from both the FeHV-1 BAC and purified virion DNA. These data showed that the FeHV-1 genome is 135,797 bp in size and has a GC content of 45%. A total of 78 open reading frames were predicted, encoding 74 distinct proteins. The gene arrangement was found to be colinear with that of most other varicelloviruses whose genomes have been sequenced [14].

All alphaherpesviruses are considered to have a replication pattern that is similar to the one of HSV-1 [6, 7]. FeHV-1 has previously been shown to contain 23 virion-associated proteins [15]. Eight glycoproteins had initially been identified, designated as gB, gC, gD, gE, gG, gH, gI, and gL. The examination of the recently derived complete sequence showed that the FeHV-1 genome in fact contains a total of 13 envelope glycoproteins [14].

Most studies on the function of FeHV-1 genes have been focused on the role of envelope glycoproteins [16], because of their predicted role in inducing protective host immune responses and, therefore, their potential for vaccine development.

## 4. Acute Infection

FeHV-1 typically affects kittens and juvenile cats. Most kittens are protected by passive immunity until they are about 2 months of age. 

The pathogenesis of FHV-1 is based upon two different mechanisms. The first is that FeHV-1 is a cytolytic virus. Examples of its cytolytic effects are ulcerations in mucosae and the cornea. The second mechanism is immune-mediated, clinically manifesting itself as stromal keratitis. An important question related to this second pathogenetic mechanism is the source of the antigenic stimulation driving this reaction [17].

The main sources of FeHV-1 transmission are oronasal and ocular secretions from acutely infected cats. Viral transmission can also be associated with the reactivation of latency. Kittens with residual passive immunity may not show clinical signs when exposed but become latently infected [18].

Following entry via the oronasal route, FeHV-1 replicates extensively in the mucosae of the upper respiratory tract and generally causes severe upper respiratory disease in susceptible animals. The incubation period varies from 2 to 6 days. The primary replication sites of FeHV-1 include the mucosae of the nasal septum, turbinate, nasopharynx, conjunctivae, and upper trachea. Replication also takes place in tonsils and mandibular lymph nodes. 

Acute respiratory FeHV-1 infection is characterized initially by fever, inappetence, and sneezing, followed by serous nasal discharge, which can become mucopurulent after 5–7 days. In addition, oral replication of the virus can result in excessive salivation and drooling of saliva. Occasionally coughing and dyspnea may occur. Oral ulceration, a typical feature of feline calicivirus infection, may occur as a result of FeHV-1 infection of the oral cavity but is uncommon [3]. 

The ocular manifestations associated with FeHV-1 infection have been reviewed by Gould [5]. In neonatal kittens ophthalmia neonatorum has been described and can lead to serious corneal damage. Acute hyperemic conjunctivitis, leading to ocular discharge and chemosis, a feature of acute infection, occurs in association with upper respiratory signs. The formation of branched epithelial ulcers, referred to as dendritic ulceration, is a pathognomonic feature of acute ocular FeHV-1 infection. In a recent review of the etiology corneal ulcers in cats, Hartley [19] stated “assume FHV-1 unless proven otherwise.” Occasionally, larger ulcers, referred to as geographic corneal ulcers, develop. Both dendritic and geographic corneal ulceration may also result from latency reactivation. Another component of lesions associated with recrudescence is conjunctival and/or corneal inflammation, which is milder than seen during acute disease.

FeHV-1 is primarily an upper respiratory and ocular pathogen, with only sporadic involvement of the lungs. Viremia levels are low, thought to be related to the natural temperature sensitivity of this virus, which would favor replication in the upper respiratory tract. Exposure of pregnant queens can lead to abortion, but infection with FeHV-1 infection is not a common cause of abortion in cats. In neonatal kittens, the infection can generalize and is associated with neurological signs and a high mortality rate. 

## 5. Alphaherpesvirus Latency Concepts

A hallmark of alphaherpesvirus biology is that acute infection is followed by lifelong persistence of the viral genome in latent form in nervous and lymphoid tissues. Latency and periodic reactivation of latency are integral parts of the lifecycle of alphaherpesviruses and important elements in their survival and transmission. 

The latency-reactivation cycle operationally consists of three major steps: establishment, maintenance, and reactivation.

The establishment of latency by definition requires that the virus reaches the tissue in which latency will be established. This process starts during the acute phase of viral replication at peripheral mucosal sites. Nerve endings of sensory nerves innervating viral replication sites take up viral particles and subparticles during this phase. These particles are transported within the axoplasm of the axons of these nerves by a process referred to as retrograde axonal transport. When the virus reaches the sensory ganglia, it infects neurons and other cell types. This acute infection of ganglionic cell types lasts for approximately one week. Neurons are the cell type in which latency is established. In order to accomplish this, lytic gene expression is repressed, while the latency-associated transcript (LAT) is expressed, which yields several RNA species by splicing. These multiple species are collectively referred to as LATs. Low level or sporadic transcription of immediate-early and early genes can occur but is not sufficient to initiate a productive infection. No infectious virions can be detected in the ganglia during latent infection. The LAT RNA is spliced, and a stable intron in the form of a lariat, called the 2-kb LAT, is produced in the nucleus. The spliced LAT mRNA is transported to the cytoplasm, where several small ORFs may be translated into proteins.

During the maintenance phase of latency, the viral DNA is present in the neurons in an episomal form. The viral DNA is not totally static during the maintenance phase of latency, but transcriptional activity of the genome is limited to a region referred to as the latency-associated transcript or LAT.

The maintenance phase of latency is reversible. In other words, under the influence of certain natural or pharmacological stimuli, the reactivation of latent viral DNA can occur. Virus replication starts up again, and infectious virions then travel back to the periphery, using the same sensory nerve “highway” used to reach the ganglia. Infectious virus can be detected again by virus isolation or PCR from nasal, oral, or ocular swabs. Usually the clinical signs associated with the reactivation process are significantly milder than those seen during the primary infection, and reactivation can certainly be asymptomatic. Virus shedding resulting from reactivation is also typically at a lower level and of shorter duration than seen during primary infection. However, reactivating virus can still be a significant source of exposure and primary disease in fully susceptible hosts that are in close contact with the animal in which reactivation took place. Reactivation occurs in only a small subset of latently infected neurons, typically less than 0.05%. Latently infected neurons in which reactivation took place do not survive. This explains why sensory deficits are not associated with reactivation in sensory nerve ganglia. Since the reservoir of latently infected neurons remains large under these conditions, repeated reactivation can take place throughout the life of the host.

Our current understanding of the regulation of latency is derived primarily from studies on HSV-1 and BoHV-1 [20–22]. The following summary is derived primarily from an excellent very recent review of HSV-1 latency by Perng and Jones [20].

### 5.1. The Role of Latency-Associated Transcripts (LATs)

Acute infection of trigeminal ganglia neurons produces toxic gene expression products that make them vulnerable to damage and death. In addition, cellular DNA damage induced by viral replication stimulates the mitochondrial pathway of apoptosis. Herpesviruses try to counteract apoptosis and thus enhance their replicative ability, by encoding several antiapoptotic genes, one of which is the LAT gene. Since there is redundancy in the viral antiapoptotic capabilities during the acute phase, apoptosis of neurons during this phase is prevented fairly efficiently. 

It is very important that apoptosis is prevented also during the establishment and maintenance stage of latency. This is especially crucial in permissive neurons, in which extensive viral replication has taken place during the acute phase. LAT exerts its antiapoptotic properties through micro-RNAs (miRNAs). A mechanism by which LAT-encoded miRNA regulates apoptosis is targeting of transforming growth factor beta, a potent inducer of apoptosis [23, 24]. 

It is important to understand the interactions between the latent viral genome and the neuron that lead to reactivation, because this is a prerequisite to ultimately controlling this process. LAT plays an important role in the *in vivo* reactivation of latency. In experimental studies it has been shown that spontaneous reactivation is severely impaired if the LAT gene is deleted. 

### 5.2. The Role of Tegument Protein VP16

Thompson et al. [25] have recently described the central role played by the tegument protein VP16 in all phases of HSV latency. Prior to establishment of latency virus replication takes place in permissive neurons. In susceptible cells at mucosal surfaces VP16, a component of virions entering the cell, combined with cellular factors, activates the immediate early genes. Axonal transport of VP16 into neurons is inefficient, which would promote latency. In order for VP16 to initiate lytic infection, it needs to be synthesized *de novo*, a process which requires that neuronal inhibition be overcome.

Very interestingly, the LAT locus is considered to express riboregulators that mediate synthesis of VP16. It has been shown that, in the absence of LAT transcription, half of the neurons destined to be latently infected instead enter the lytic cycle and die. In contrast when repression is overcome, neurons become lytically infected, and the infectious virus produced spreads both within the ganglia and back to the mucosal surface where infection was initiated. The goal of lytic infection is to increase the number of latently infected cells.

Stress, leading to reactivation, is hypothesized to increase the novo production of VP16 by a mechanism that is still under investigation. The VP16 produced then initiates a feedback loop with the IE genes and results in viral reactivation in a very limited number of latently infected neurons. 

### 5.3. The Role of Local Cell-Mediated Immune Responses

T cells, especially CD8+ T lymphocytes, have been found to be crucial for acute controlling HSV infection in sensory ganglia. Viral antigen production in trigeminal ganglia increases until 3 days after infection but is no longer detectable at 7 days after infection. As antigen production decreases, there is an increase of different types of different types of lymphoid cells, such as macrophages, natural killer cells (NK), and certain CD8+ T cells surrounding infected neurons. 

It is thought that T cells, especially CD8+ T lymphocytes, inhibit reactivation from latency. Persistence of immune effector cells in trigeminal ganglia (TG) implies that low levels of viral proteins are expressed and that an immune response occurs. In a mouse HSV-1 model, it has been demonstrated that viral DNA replication, transcription, and viral protein production take place in 1 neuron per 10 TG. These individual neurons are considered to be undergoing “spontaneous molecular reactivation” and are consistently surrounded by cuffs of infiltrating white blood cells. Two mechanisms by which these infiltrating cells prevent reactivation are the production of gamma interferon and lymphocyte-mediated cytotoxicity. 

## 6. Reactivation of Latency

The trigeminal ganglion is considered a primary site of latency for FeHV-1 although recent studies implied other tissues as potential sites [26, 27].

Spontaneous reactivation is possible but does not occur frequently. More commonly leading to the reactivation of latent FeHV-1 is the result of environmental or physiological stresses, such as changes in housing or lactation. The reactivation frequency rates have been reported to be 18% as a result of moving cats to a new environment and 40% as a result of lactation [28–30]. The lag phase between the stressor leading to reactivation and the actual shedding of infectious virus is about 4–11 days, and virus excretion lasts for approximately 6 days on average. Virus excretion by cats in which a reactivation event took place ranges from 1–13 days [29, 31]. During this time infectious virus can be demonstrated in ocular and oronasal secretions. The reactivation can be either asymptomatic or associated with clinical signs. Symptomatic reactivation is referred to as recrudescence. Reactivation of latent viral DNA in adult cats can lead to corneal ulceration, accompanied by varying degrees of conjunctivitis [32]. Since herpetic stromal keratitis caused by HSV-1 is the leading cause of infectious blindness in industrialized countries, ocular infection of FeHV-1 in cats is considered a very good natural host model.

The administration of corticosteroids has been reported to lead to reactivation in 70% of the latently infected cats [3]. Infectious virus is carried by anterograde axonal transport to peripheral tissues, usually to cells at or near the site of initial infection, and is a potential source of viral transmission [6, 7].

The role of reactivation in the epidemiology of alphaherpesviruses is directly related to the frequency by which it takes place. Some herpesviruses, including FeHV-1, reactivate much more easily than others from the latent state, both under natural and experimental conditions. The ease by which latent FeHV-1 DNA is reactivated is an important element in the justification of FeHV-1 infection of cats as a natural host model to study the molecular pathogenesis of herpesvirus latency and approaches to prevent it.

## 7. Diagnosis 

Clinically, there is an overlap between the symptomatology of acute FeHV-1 and feline calicivirus (FCV), another major respiratory disease of cats. Distinguishing features of FeHV-1 infection are high fever and corneal ulcerations. In contrast, ulcers of the tongue, palate, and pharynx are more typical or encountered more frequently in calicivirus infections.

The most common laboratory diagnostic methods to demonstrate the presence of FeHV-1 or viral components in tissue homogenates or swabs include the direct fluorescent antibody (FA) test, virus isolation (VI), and PCR [3, 5, 18]. 

Fluorescent antibody testing is performed on conjunctival or corneal tissue. This test is far less commonly used now than it used to be. Topical fluorescein, used to visualize ulcers, should be avoided prior to collecting samples.

Laboratory diagnosis of acute FeHV-1 is now most commonly performed by virus isolation (VI) or PCR, using oronasal and conjunctival swab extracts as the samples. VI detects infectious virus and has been the laboratory diagnostic gold standard [4, 28]. 

Multiple PCR assays have been described for use in the detection of FeHV-1 DNA. An excellent TaqMan-based real-time PCR assay, described by Vögtlin et al. [33], targets a conserved portion of the FeHV-1 gB gene. The assay was determined to be very specific for FeHV-1, and its detection limit was between 0.6 and 6TCID_50_. Infectious virus titers and viral DNA correlated over a wide dilution range. The real-time PCR (qPCR) was evaluated on sequentially collected ocular fluid extracts. Early during infection, referred to as phase 1, the correlation between virus titers and qPCR signals was very high. Next, during so called phase 2, a rapid decline in infectious virus titers was seen, while the qPCR signals remained high. During the final phase, referred to as phase 3, infectious virus was no longer detectable, and the quantitative PCR signals were also declining. Analysis of the combined virus detection and qPCR results on 20 clinical samples allowed the authors to reliably define the phase of the infection during which the samples had been collected. Realizing the cost of combined testing, it was suggested to test consecutive samples by qPCR to accomplish this goal. 

Maggs [4] pointed out 3 aspects of laboratory diagnosis of FeHV-1 that can be very frustrating for the clinician. Whereas the confirmation of acute FeHV-1 is not always required, it is important to confirm that chronic lesions are caused by FeHV-1. Unfortunately, the detection of FeHV-1 or viral components in these lesions can be difficult. The second aspect of laboratory diagnosis that leads to misinterpretations is the fact that FEHV-1 or viral l DNA can be detected in samples from clinically normal cats. It was pointed out that the detection of FeHV-1 or its components can be coincidental, consequential, or causal. Differentiating between these possibilities is obviously important.

Virus neutralizing antibody titers are determined by VN tests, which are commonly used to detect prior infection or the efficacy of vaccination. Virus neutralizing antibodies can be low and slow to develop. As pointed out by Dawson et al. [34], a low level of neutralizing antibodies does not imply the absence of protection against clinical disease.

## 8. Treatment and Control

### 8.1. Supportive Treatment

Guidelines for the management of FeHV-1-induced disease have been published by The European Advisory Board on Cat Diseases (ABCD) [18]. As is the case for many viral infections, supportive therapy is being advised. Broad spectrum antibiotics that achieve good penetration into the respiratory tract should be administered in all acute cases to prevent secondary bacterial infections. Intake of food that is palatable and flavorful is also important, since infected cats develop anorexia from the loss of their sense of smell or, less commonly, the presence of ulcers in the oral cavity. In cats with severe clinical signs, the restoration of fluids, electrolytes, and acid-base balance is required, preferably intravenously. Nasal decongestants, mucolytic drugs, and nebulization with saline can all ameliorate clinical signs. Eye drops or ointments, when used, should be administered several times a day.

### 8.2. Antiviral Therapy

Antiviral therapy consists of topically or systemically administered antivirals or the use of adjunctive therapies. Comparison of 8 antiviral drugs administered topically demonstrated that the highest efficacy was obtained with trifluridine, based upon its potency and corneal penetration. Second in effectiveness was idoxuridine, which has a lower cost and appears to be less irritating [4]. 

Nucleoside analogue antivirals are commonly used to treat HSV and VZV infections. They are converted into triphosphates by viral thymidine kinase and other host enzymes in infected cells and competitively inhibit viral DNA polymerase. This prevents DNA chain elongation [35] and, as a result, disrupts viral replication.

The use of these agents against FeHV-1 infection has been largely limited to topical administration. First generation nucleoside analogues, including acyclovir and its prodrug valacyclovir, have little efficacy against FeHV-1 *in vitro *and moderate effect *in vivo*. More importantly, when administered systemically they produce serious side effects in cats, including myelosuppression, hepatotoxicity, and nephrotoxicity at therapeutic levels [36, 37]. 

According to the guidelines of the European Advisory Board for Cat Diseases (ABCD), trifluridine is the topical treatment of choice in cats with ocular FHV-1 manifestations. Acyclovir, ganciclovir, and idoxuridine are also suggested for topical use. It was noted that, except for acyclovir, there is a lack of controlled *in vivo *efficacy study for these agents in the literature [18]. The efficacy of topical application of cidofovir on primary ocular FeHV-1 infection has been demonstrated [38]. 

Although the study wasn't controlled, oral administration of famciclovir has been reported to be safe and efficacious in treating ocular signs, cutaneous disease, and rhinosinusitis induced by FeHV-1 infection [39]. 

Adjunctive therapies that are used to treat FeHV-1 infection are L-lysine, lactoferrin, and interferons. L-lysine is an antagonist of arginine; the latter has been shown to be essential for HSV-1 and FeHV-1 protein synthesis [40]. Treatment with L-lysine, therefore, decreases viral replication and has been shown to have some inhibitory effect against both human herpesvirus and FeHV-1 infection. An issue with low dietary arginine concentrations is the pronounced susceptibility of cats to arginine deficiency [40, 42].

Oral supplementation with L-lysine reduces the severity of experimentally induced FeHV-1 conjunctivitis [42] and ocular virus shedding associated with the reactivation of latent infection [40]. It was suggested for use early in acute disease or as a means of reducing the severity of disease and virus shedding at times of stress [3]. It has been demonstrated that L-Lysine is safe at relatively high oral dose levels. 

Lactoferrin is a mammalian iron-binding glycoprotein. It has been shown [43] to inhibit FeHV-1 replication *in vitro, *potentially as a result of interfering with the binding of FeHV-1 binding to its cellular receptor and/or viral penetration into susceptible cells. 

Interferons are cytokines released by white blood cells and interfere with viral cell-to cell spread. Interferon-alpha (IFN-*α*) administration has been shown to decrease clinical signs associated with acute infection [3].

## 9. Immunity and Vaccination

Primary FeHV-1 infection induces both humoral and cellular immune responses. Active immunity induced by natural FeHV-1 infection or immunization protects cats from the disease, but not from infection. Mild clinical signs have been observed upon reexposure as soon as 150 days after the primary infection [18, 44, 45]. Virus neutralizing antibody titers are generally low and in some cases undetectable after primary infection; although after further exposure to virus, they tend to rise to more moderate levels and thereafter remain reasonably stable [3, 46]. Since FeHV-1 targets the eye and upper respiratory tract, mucosal immune responses also play a significant role [47].

Passive immunity persists for 2 to 10 weeks, depending upon colostrum concentration and intake. Some kittens with low levels of maternally derived antibodies that are exposed to field virus may develop subclinical infection and latency [48]. Alternatively, such kittens would also respond to early vaccination. Conversely, in some kittens maternally derived antibodies are high enough to still be at interfering levels at 12–14 weeks of age [3, 49]. 

Vaccination recommendations have been provided by The European Advisory Board for Cat Diseases (ABCD) and The American Association of Feline Practitioners Feline Vaccine Advisory Panel.

The ABCD panel recommends an initial two-dose vaccination regimen: the first dose being given at 9 weeks of age and the second at 12 weeks of age. This is followed by yearly boosters [18].

The American Association of Feline Practitioners Feline Vaccine Advisory Panel advises that the primary immunization dose should be given as early as 6 weeks of age, with additional doses every 3 to 4 weeks until 16 weeks of age. A booster dose is to be administered 1 year following the last dose of the primary series. Subsequent booster doses are then administered every 1–3 years [50].

All current commercial vaccines against FVR also contain feline calicivirus (FCV) and feline panleukopenia virus (FPV) components and are collectively termed FVRCP vaccines. The protection induced by these trivalent vaccines is generally the lowest against the FeHV-1 component [51, 52].

Both modified-live and inactivated FVRCP vaccines for systemic use are available in the United States [50, 53]. Modified-live vaccines (MLVs) are routinely used, but they have residual virulence and may induce clinical signs if administered incorrectly [54]. Because of safety concerns, inactivated vaccines are mostly preferred for use in pregnant queens, and in cats that are infected with feline leukemia virus (FeLV) or feline immunodeficiency virus (FIV) [50].

In addition to vaccines labeled for systemic immunization, an intranasal multivalent vaccine containing a FeHV-1 component is commercially available. Testing under experimental conditions showed that this vaccine was safe and induced protection against the clinical signs of field virus exposure within a week after vaccination [53], versus 2-3 weeks with a systemically administered vaccine [55].

## 10. New Approaches to Immunization

### 10.1. Virulence Genes and Deletion Mutant Vaccines

As discussed earlier, currently available vaccines cannot totally protect cats from field virus infection and, as a consequence, from field virus latency [56–60]. 

A better understanding of herpesvirus virulence factors is a prerequisite for the generation of safe and efficacious deletion mutant vaccines. Candidate genes for deletion are those encoding the nonessential glycoproteins gC, gE, and gG, the US3 gene encoding a protein kinase, the UL 23, gene encoding thymidine kinase. The combination of BAC cloning of herpesvirus genomes and the introduction of recombineering to rapidly generate mutants within alphaherpesviruses cloned as BACs have been very useful tools to generate mutants with vaccine potential. 

Glycoprotein E (gE) is a virulence factor of FeHV-1. Glycoprotein E (gE) and glycoprotein I (gI) form a heterodimer that functions in virus cell-to-cell spread of the virus and transsynaptic spread of infection throughout the host nervous system, an important component of neurovirulence. gE/gI are nonessential glycoproteins, except for MDV [61]. As an *in vitro* indicator of reduced virulence, gE/gI mutants have a smaller plaque size and reduced capacity for cell-to-cell spread [62–66]. A functional gE/gI heterodimer appears to play an even greater role in the spread of VZV [67–69].

We previously constructed a gE/gI deletion mutant by conventional *in vivo* recombination and reported that cats vaccinated subcutaneously with high doses of the recombinant FeHV-1 strain responded with only mild clinical signs and developed strong immunity against subsequent virulent virus challenge [70]. We also compared the intranasal and subcutaneous routes of administration of this strain and assessed its ability to induce protective immunity and prevent virus shedding after challenge. The only concern we had is that this mutant had some residual virulence when administered intranasally at high dosage levels [54].

Kaashoek et al. [71] constructed gE-, TK-, and gE-TK-deletion mutants of BoHV-1 and examined their virulence and immunogenicity in calves. After intranasal inoculation, the TK mutant showed some residual virulence, whereas the gE and gE-TK mutants were completely avirulent. The calves inoculated with these deletion mutants were protected against clinical disease after challenge exposure and shed significantly less challenge virus than control calves.

Recently, an EHV-1 gE mutant was evaluated as a modified live virus (MLV) vaccine. Colostrum-deprived foals inoculated intranasally (IN) or intramuscularly (IM) with the gE mutant did not exhibit any clinical signs of respiratory disease except for mild nasal discharge in one of the IN inoculated foals on Days 1 and 3 after infection. In contrast, foals inoculated IN with the revertant had biphasic fever, mucopurulent nasal discharge, and submandibular lymph node swelling. The efficacy of the gE mutant against wild type EHV-1 challenge infection was assessed using foals previously vaccinated twice IM with 10^5^ or 10^6^ plaque-forming units (pfu) of the gE-mutant at an interval of 3 weeks. These foals exhibited no respiratory disease signs after IM immunization and developed a good virus neutralizing antibody response to EHV-1 after the second dose. Following a wild-type EHV-1 challenge infection, vaccinated foals showed milder clinical symptoms than foals vaccinated with a placebo, and challenge virus shedding was significantly reduced [72].

The thymidine kinase (TK) gene of alphaherpesviruses is a virulence factor. Comparisons of the amino acid sequences of herpesvirus TK proteins showed that these proteins are highly divergent, sharing only short regions of imperfect amino acid identity. Nunberg et al. [73] first identified the TK gene of FeHV-1 using PCR with highly degenerate oligonucleotide primers. Yokoyama et al. [74] inserted the gene encoding the feline calicivirus capsid protein into the TK locus of FHV-1 and designated the recombinant C730ldfTK-Cap. In a pilot study, 2 cats were inoculated intranasally and orally with C730ldfTK-Cap, and one cat was inoculated via the same routes with C730ldfTK. Virus-neutralizing (VN) antibody against both FeHV-1 and FCV was induced with C730ldfTK-Cap, and against FeHV-1 with C730ldfTK.

The US3 gene of FeHV-1 encodes a serine/threonine protein kinase (PK), and its amino acid sequence is conserved in the subfamily *Alphaherpesvirinae* [75–77]. Possible functions of PK include blocking of apoptosis induced by both viral and cellular proteins [78–81], regulation of the nuclear egress of progeny nucleocapsids [82, 83], and control of the morphology of infected cells [84, 85]. Kimman et al. [86] demonstrated that a PK-mutant of pseudorabies virus (PRV) has strongly reduced virulence, and animals inoculated with PK-gE-PRV mutant and subsequently challenged with wild-type virus has reduced virus shedding.

Glycoprotein C (gC) homologues have been extensively studied in several alphaherpesviruses. gC homologues are nonessential for herpesvirus replication *in vitro*, but they mediate several important biological functions. First of all, gC is involved in the initial step of viral attachment by interacting with heparan sulfate on cell surface, as demonstrated in HSV-1, PRV, BHV-1, and EHV-1 [87–90]. gC deficient mutants attach to cells with reduced efficiency [90]. Secondly, gCs of HSV-1 and -2 can bind the complement component C3b [91, 92]. Binding of this complement factor may protect herpesvirus-infected cells from complement-mediated lysis [93]. Viruses lacking complement-binding domains are less virulent than wild-type virus [87, 91, 92]. The gC of FeHV-1 has been shown to be the dominant heparin-binding glycoprotein that mediates the initial stage of viral adsorption, as observed in other herpesviruses [94]. However, it remains to be determined whether FeHV-1 gC protects virus-infected cells from complement-mediated lysis.

Willemse et al. [95] first determined a partial sequence of gC. They also found that the adjacent UL45 gene can be cotranscribed with gC. The complete sequence of FeHV-1 gC was later determined by Maeda et al. [96]. Based on the amino acid sequence deduced from the nucleotide sequence, they predicted that gC is a membrane glycoprotein containing a characteristic N-terminal hydrophobic signal sequence, nine potential N-linked glycosylation sites, and C-terminal transmembrane and cytoplasmic domains. Maeda et al. [97] further demonstrated that gC is the major heparin-binding glycoprotein involved in the initial step in virus adsorption to cells as observed in gCs of other herpesviruses. In addition, they found that gC can agglutinate murine red blood cells, and that infection of FeHV-1 is inhibited by the addition of soluble heparin in cells cultures. 

The gG glycoprotein of herpesviruses interacts with chemokines, which are involved in the regulation of leukocyte trafficking and function and the regulation of inflammation and immunosurveillance. The gG glycoprotein of alphaherpesviruses can exist in three different forms: membrane-bound full length, membrane bound truncated or secreted. The full length form, present in FeHV-1 and EHV-1, can also exist as a truncated secreted form. The secreted form functions as a viral chemokine-binding protein (vCKBP) and is now classified under the vCKBP-4 subfamily [98].

Van de Walle et al. [99] used EHV-1 as a model to provide the first molecular determination of the residues in gG of EHV-1 involved in chemokine binding and interaction with target cells. In a very recent study, Thormann et al. [100] constructed recombinant viruses to show that the ability of the gG of EHV-1 to interfere with chemokine is not entirely mediated by its chemokine- binding region.

The gG of FeHV-1 exists in booth membrane-bound and secreted forms. The secreted form shows *in vitro* binding to bind to a number of chemokines. The membrane bound displays true viroreceptor characteristics [101]. 

Virulence characteristics of the gG of several alphaherpesviruses have been investigated. It has been shown previously that the deletion of gG in PRV does not have a significant effect on viral virulence [102]. In contrast, the administration of a gG-deleted ILTV to birds, the natural host of this virus, showed that the gG deletion resulted in significant reduction in virulence. Importantly, virulence could be restored with a revertant, and the transcription of genes adjacent to the gG deletion was not affected by the gG deletion. Immunization with the gG deletion mutant was shown to be protective against virulent virus challenge in experimental birds [103–105]. 

Herpesviruses have multiple immune evasion genes with various evasion mechanisms.

UL49.5 is a gene present in the genome of several members of the *varicellovirus* genus, such as EHV-1, BoHV-1, and PRV. UL49.5 inhibits transporter associated with antigen processing (TAP) and downregulates cell-surface expression of major histocompatibility complex (MHC) class I molecules [106]. 

A BoHV-1 UL49.5 null mutant was shown to no longer have the TAP inhibition and MHC-I downregulation properties of the parent virus [107]. In a follow-up study, the pathogenicity and immune responses in calves infected with BoHV-1 UL49.5 null mutant and the parent wild type strain were compared. Both strains replicated similarly in the nasal epithelium, and both groups had similar clinical scores. BoHV-1 antigen-specific CD8+ T-cell proliferation as well as CD8+ T-cell cytotoxicity in calves infected with the BoHV-1 UL49.5 null mutant peaked by 7 days after infection, 1 week earlier than in calves infected with the wild type strain. In addition, virus neutralizing antibody (VN) titers and IFN-*γ*-producing peripheral blood mononuclear cells (PBMCs) in the UL49.5 mutant virus-infected calves also peaked 7 days and 14 days earlier, respectively. This study indicated that while immune responses peak earlier, deleting UL49.5 by itself did not sufficiently attenuate this alphaherpesvirus to make it a vaccine candidate [108].

### 10.2. Generating Mutants by BAC Clone Recombineering

BAC cloning and recombineering are two state-of-the-art techniques to facilitate the process of mutagenesis. BACs are single copy F-factor-based plasmid vectors, which can stably hold 300 kb or more of foreign DNA [109]. The BACs' larger capacity and greater stability over the other vectors have enabled the cloning of an entire herpesvirus genome into a single plasmid. These properties have also made BAC the vector of choice for the cloning of herpesvirus genomes.

Recombineering is a powerful method for fast and efficient manipulation of the BAC. It allows DNA cloned in *E. coli* to be modified via lambda (*λ*) red-mediated homologous recombination, obviating the need for restriction enzymes and DNA ligases. Specific bacterial strains, for example, *E. coli* SW105, have been constructed for this purpose [110–112]. A defective *λ* prophage (mini-*λ*) is inserted into the *E. coli* genome and encodes heat-shock inducible genes that make recombineering possible. Linear DNA (PCR product, oligonucleotide, etc.,) with sufficient homology in the 5′ and 3′ ends to a target DNA molecule already present in the bacteria (plasmid, BAC, or the bacterial genome itself) can be electroporated into heat-shocked and electrocompetent bacteria cells and undergoes homologous recombination with the target molecule. Utilizing recombineering techniques, site-specific mutations can be introduced anywhere in the viral genome. All mutagenesis steps can be strictly controlled and analyzed in *E. coli*, and the manipulated viral genome can be stably maintained in the *E. coli*.

The entire FeHV-1 genome was previously cloned as a BAC in our lab, from which the complete FeHV-1 genomic sequence was derived [14]. The BAC-cloned virus was characterized *in vitro* and *in vivo*. Prior to defining the *in vitro* growth characteristics of the BAC-cloned virus, the BAC cassette was excised from the cloned virus genome. We then performed plaque size analysis and constructed multiple-step growth curves for the FeHV-1ΔBAC and its C-27 parent strain. Plaques produced by the C-27 strain and FeHV-1ΔBAC virus were morphologically undistinguishable from each other. The mean plaque diameter of the FeHV-1ΔBAC virus was 101.05% of that of the C-27 parent strain and not significantly different. Multistep growth curve analysis showed that they can grow to a similar titer.

To investigate possible attenuation resulting from BAC cloning itself, a preliminary challenge experiment was carried out, using four specific-pathogen-free (SPF) cats. Two cats were inoculated intranasally with the FeHV-1ΔBAC virus, and the other two cats were inoculated intranasally with either the C-27 strain or cell culture medium. The main conclusion from the *in vivo *experiment was that the BAC clone-derived virus behaved very similarly to its C-27 parent strain both *in vitro* and *in vivo*, making it an excellent starting platform for introducing mutations aimed at deleting virulence-inducing genes from the FeHV-1 genome [14].

## 11. Mucosal Vaccination and Epitope-Based Vaccines

A major goal of strategies to immunize against alphaherpesvirus infections is to prevent primary infection, which would in turn prevent primary disease and the establishment of latency and subsequent latency reactivation. Latency reactivation has been shown to occur frequently, leading to virus shedding, which is asymptomatic in most cases. Natural infection provides protection against reinfection of primary mucosal replication sites for a certain period of time. This provides a rationale for the development of immunization strategies at the mucosal level. 

Innate immune responses are the first to develop after natural infection. The recognition of alphaherpesvirus components by toll-like receptors is an important mechanism for induction of these responses. HSV and its components bind to TLR 2, 3, 7, and 9. Synthetic agonists have been designed to transiently activate the innate immune response [113].

In human medicine, the majority of the efforts to develop immunization strategies against herpesvirus infections have been focused on the prevention of genital herpes. However, it is also well recognized that ocular HSV-1 infection is a leading worldwide cause of herpetic keratitis, which can lead to corneal blindness. Like is the case for FeHV-1, the most severe ocular HSV-1 infections are the result of repeated reactivation events. It is clear that mucosal delivery is the best approach to generate secretory immunity and cytotoxic T-cell responses at mucosal sites.

Long-term efforts to immunize against human alphaherpesvirus infections have included subunit vaccines, modified-live vaccines, replication-defective vaccines, viral vector vaccines, and naked DNA vaccines. Despite these efforts, there are no licensed vaccines available.

One of the current approaches to mucosal immunization focuses on the development of a multiepitope self-adjuvant lipopeptide vaccine. A recent overview of this approach by the group that has pioneered it highlights its promise, but also the hurdles that still have to be overcome [114]. They point out that, based upon recent trials, the induction of neutralizing antibodies is not sufficient for protection. Implied from these results is that the induction of appropriate and adequate protective T-cell responses is a crucial part of the development of protective immunity. The essential components of a protective immune response can be the prevention of primary infection or the prevention or reduction of reactivation events. It is clear from their work, and that of others, that individuals that are latently infected with HSV, have frequent reactivation events associated with virus shedding. This reactivation is not associated with clinical signs in most individuals, which are therefore termed asymptomatic individuals. In contrast, the term symptomatic individuals is used for those in which frequent reactivation is associated with clinical signs. An important element of the strategy is to characterize the unique T-cell repertoire in HSV-positive individuals that do not suffer from frequent symptomatic reactivation. It has been determined that a set of human T-cell epitopes from HSV-1 gB and gD are strongly recognized by T-cells from asymptomatic individuals, but not by T cells from symptomatic individuals. In contrast, another nonoverlapping set of gB and gD epitopes is recognized by symptomatic individuals. The results of recent immunization of asymptomatic HLA transgenic rabbits showed that immunization with asymptomatic CD8+ epitopes from HSV-1 gD induced strong CD8+ immune responses and reduced HSV-1 shedding and tears and corneal lesions following ocular challenge virus administration.

The authors emphasize repeatedly that the following five existing hurdles need to be overcome: (1) reasons for suboptimal immunity resulting from natural infection, (2) optimal effector mechanisms for protective immunity against the acute and latent phases of the disease, (3) knowledge about immunoevasive strategies, (4) distinction between protective versus pathogenic antigens, and (5) design of a an appropriate vaccine delivery system. They recognize that candidate vaccines need to be tested in relevant animal models if they cannot be directly evaluated in the natural host. An already existing human HLA transgenic rabbit model and the development of a similar guinea pig model are crucial tools in this respect. 

## 12. *In Vitro* Approaches to Molecular Pathogenesis

Ocular infection with FeHV-1 results from viral exposure of conjunctival and corneal tissue. Since corneal lesions are an important disease manifestation, both during both the acute phase and as a result of reactivation, finding an effective therapy against development of ocular disease has high priority. Sandmeyer et al. [115] have reported the development of primary corneal cell culture system, which is useful for *in vitro* pathogenesis of ocular disease and also for the testing of potential antivirals [116]. Using this system they showed that IFN-*α* was not toxic to ocular cells and had a limited effect of virus production in FeHV-1-infected corneal cells. They speculated that a combination of IFN-*α* and other antivirals may act synergistically [117].

Pathogenesis studies of FeHV-1 have almost exclusively been done on live animals. Since the tracheal mucosa is an important replication site of FeHV-1, tracheal organ cultures are a good *in vitro* model to study viral invasiveness and local immune responses. Leeming et al. [118] established feline tracheal organ cultures and showed that these could be maintained for at least 5 days. Infection of these cultures at different multiplicities of infection (MOI), ranging from 0.1 to 100, showed that the virus replicated extensively in these cultures and produced coalescing necrosis of tracheal epithelium and disruption of ciliary activity. 

Since mucosal surfaces are the primary replication sites of FeHV-1, it is important to understand viral replication strategies and the local immune responses generated at these sites to better combat this mucosal pathogen. As indicated earlier, it is well known that systemically administered vaccines can prevent clinical signs but cannot prevent reinfection and the associated development of latency.

Quintana et al. [119] recently developed an equine respiratory epithelium cell culture system consisting of culturing dissociated primary epithelial cells at a liquid air interface. This is a meaningful *in vitro* system since epithelial cells not only provide a physical barrier against viral invasion, but also play a significant role in development of immunity by expressing toll-like receptors, by secreting cytokines, chemokines, and host defense peptides and by playing some role in antigen presentation. It was shown that epithelial cell cultures grown under these conditions were morphologically similar to intact airway epithelium. These cultures were also shown to be immunologically competent, but some properties were altered by *in vitro* culture under sterile conditions. The authors concluded that the addition of antigenic stimuli and/or immune cells could reverse this situation.

Mucosal explants have recently been shown for several herpesviruses to be an excellent system to study kinetics of viral invasion, as determined by the ability of a particular herpesvirus to get across the epithelial basement membrane. This system has been used to compare the invasiveness of different herpesviruses. It can, however, also be used for strain comparison and to study the role of individual or combinations of viral genes as determinants of viral virulence [120–123].

FeHV-1 infection of cats is an excellent natural host model to study mechanisms involved in establishment, maintenance, and reactivation of latency. As discussed above, latency is established in all cats following natural infection and is readily reactivated by a variety of natural stimuli or administration of corticosteroids.

De Regge et al. [124] reported the development of a homologous *in vitro* model to study the interaction of alphaherpesviruses and trigeminal ganglion neurons. The system consists of two concentric culture chambers. The inner and outer chambers are separated from one another by a silicon barrier, which is impermeable to both virus and cell culture medium. After 2-3 weeks in culture, axons from neuronal cell bodies present in the inner chamber grow through the silicon barrier into the outer chamber. Infection of these axons, either with HSV-1 or PRV, exclusively led to infection of neurons in the inner chamber and the subsequent spread of infection from these neurons to other neurons and nonneuronal cells. This system thus allows an *in vivo*-like infection of neuronal cells via retrograde axonal transport. It is, therefore, very useful to study mechanisms involved in latency establishment, maintenance, and reactivation. In a follow-up study, De Regge et al. [125] used this system to examine the role of IFN-*α*, an important component of the innate immune system. The data showed that IFN-*α* was indeed able to establish latency in these cultures and that latency was maintained after its removal. LAT transcripts, a prominent feature of latency, were detected in the cultures by RT-PCR and the latent viral DNA could be reactivated by treatment with forskolin. 

## 13. A New Approach to Antiviral Therapy

Ribonucleic acid interference (RNAi), initiated by chemically synthesized 21-mer or 27-mer small interfering RNAs (siRNA), is an alternative method to the use of standard antiviral therapy. Wilkes and Kania [126] have explored the potential of this method *in vitro*. The initial target was gD-specific mRNA, based upon the fact that the gD glycoprotein plays an important role in viral attachment to susceptible cells and also in the induction of protective neutralizing antibody responses. Two of the six siRNAs they tested induced a significant reduction of virus replication in CRFK cells infected with FeHV-1. In a follow-up study Wilkes and Kania [127] selected siRNAs specific for the FeHV-1 DNA polymerase mRNA, the gD mRNA, or a combination of both. The hypothesis behind the targeting of the DNA polymerase was that more complete inhibition of viral replication would occur when an early rather than a late transcript was targeted. This proved to be the case, since the highest level of inhibition was obtained with a combination of 2 siRNAs targeting the FeHV-1 DNA polymerase transcript. Potential *in vivo* use of this approach is based upon the fact that siRNAs can be taken up effectively when applied to mucosal surfaces. 

## 14. Conclusions

Recent expansion in our molecular knowledge of FeHV-1 will ultimately not only be of benefit to the health of domestic cats but will also contribute to our understanding of shared aspects of herpesvirus biology. FeHV-1 infection in cats also has potential, as a natural host system, to develop more effective immunization and treatment procedures against alphaherpesvirus infections in animals and humans.
